# Influenza Virus Transmission Is Dependent on Relative Humidity and Temperature

**DOI:** 10.1371/journal.ppat.0030151

**Published:** 2007-10-19

**Authors:** Anice C Lowen, Samira Mubareka, John Steel, Peter Palese

**Affiliations:** 1 Department of Microbiology, Mount Sinai School of Medicine, New York, New York, United States of America; 2 Department of Medicine, Mount Sinai School of Medicine, New York, New York, United States of America; University of North Carolina, United States of America

## Abstract

Using the guinea pig as a model host, we show that aerosol spread of influenza virus is dependent upon both ambient relative humidity and temperature. Twenty experiments performed at relative humidities from 20% to 80% and 5 °C, 20 °C, or 30 °C indicated that both cold and dry conditions favor transmission. The relationship between transmission via aerosols and relative humidity at 20 °C is similar to that previously reported for the stability of influenza viruses (except at high relative humidity, 80%), implying that the effects of humidity act largely at the level of the virus particle. For infected guinea pigs housed at 5 °C, the duration of peak shedding was approximately 40 h longer than that of animals housed at 20 °C; this increased shedding likely accounts for the enhanced transmission seen at 5 °C. To investigate the mechanism permitting prolonged viral growth, expression levels in the upper respiratory tract of several innate immune mediators were determined. Innate responses proved to be comparable between animals housed at 5 °C and 20 °C, suggesting that cold temperature (5 °C) does not impair the innate immune response in this system. Although the seasonal epidemiology of influenza is well characterized, the underlying reasons for predominant wintertime spread are not clear. We provide direct, experimental evidence to support the role of weather conditions in the dynamics of influenza and thereby address a long-standing question fundamental to the understanding of influenza epidemiology and evolution.

## Author Summary

In temperate regions influenza epidemics recur with marked seasonality: in the northern hemisphere the influenza season spans November to March, while in the southern hemisphere epidemics last from May until September. Although seasonality is one of the most familiar features of influenza, it is also one of the least understood. Indoor crowding during cold weather, seasonal fluctuations in host immune responses, and environmental factors, including relative humidity, temperature, and UV radiation have all been suggested to account for this phenomenon, but none of these hypotheses has been tested directly. Using the guinea pig model, we have evaluated the effects of temperature and relative humidity on influenza virus spread. By housing infected and naïve guinea pigs together in an environmental chamber, we carried out transmission experiments under conditions of controlled temperature and humidity. We found that low relative humidities of 20%–35% were most favorable, while transmission was completely blocked at a high relative humidity of 80%. Furthermore, when guinea pigs were kept at 5 °C, transmission occurred with greater frequency than at 20 °C, while at 30 °C, no transmission was detected. Our data implicate low relative humidities produced by indoor heating and cold temperatures as features of winter that favor influenza virus spread.

## Introduction

Influenza A virus, of the family Orthomyxoviridae, carries an RNA genome consisting of eight segments of negative-stranded RNA. This genome encodes one or two non-structural proteins and nine structural proteins, which, together with a host cell–derived lipid envelope, comprise the influenza virus particle. Influenza virus causes widespread morbidity and mortality among human populations worldwide: in the United States alone, an average of 41,400 deaths and 1.68 million hospitalizations [1] are attributed to influenza each year. In temperate regions like the United States, this impact is felt predominantly during the winter months; that is, epidemics recur with a highly predictable seasonal pattern. In northern latitudes, influenza viruses circulate from November to March, while in the southern hemisphere influenza occurs primarily from May to September [2]. Tropical regions, by contrast, experience influenza throughout the year, although increased incidence has been correlated with rainy seasons [2,3]. Despite extensive documentation of the seasonal cycles of influenza and curiosity as to their causes, little concrete data is available to indicate why influenza virus infections peak in the wintertime. Theories to explain the seasonal variation of influenza have therefore proliferated over the years (reviewed in [4]). Current hypotheses include fluctuations in host immune competence mediated by seasonal factors such as melatonin [5] and vitamin D [6] levels; seasonal changes in host behavior, such as school attendance, air travel [7], and indoor crowding during cold or rainy weather; and environmental factors, including temperature [8], relative humidity (RH), and the direction of air movement in the upper atmosphere [9]. In early studies using mouse-adapted strains of influenza virus, experiments performed in the winter months yielded a transmission rate of 58.2%; in contrast, a rate of only 34.1% was observed in the summer months [10]. While these data suggested that the seasonal influences acting on humans also affect laboratory mice, no mechanism to explain the observations was identified.

Herein, we directly tested the hypotheses that ambient air temperature and RH impact the efficiency with which influenza virus is spread. As a mammalian animal model we used Hartley strain guinea pigs, which we have recently shown to be highly susceptible to infection with human influenza viruses [11]. Importantly, we also found that naïve guinea pigs readily become infected when exposed to inoculated guinea pigs, unlike mice, which do not efficiently transmit influenza virus [11]. Thus, by housing infected and naïve guinea pigs together in an environmental chamber, we were able to assess the efficiency of transmission under conditions of controlled RH and temperature. Our data show that both RH and temperature do indeed affect the frequency of influenza virus transmission among guinea pigs, although via apparently differing mechanisms.

## Results

**Figure 1:**
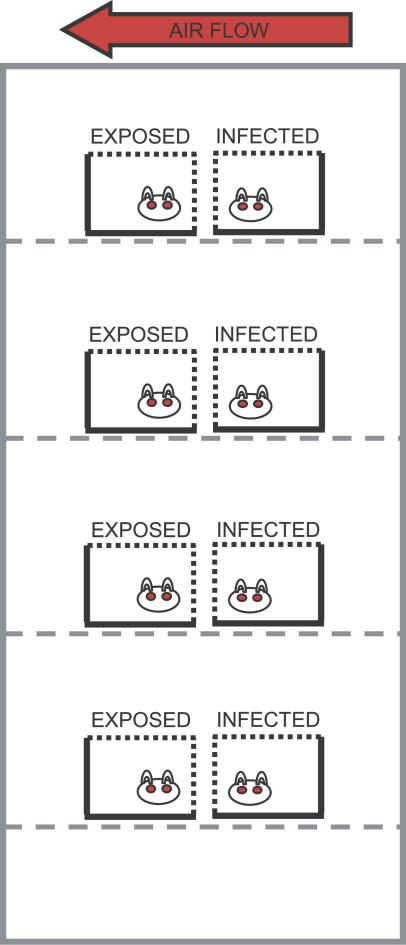
Arrangement of Infected and Exposed Guinea Pigs in Environmental Chamber In each experiment, eight animals were housed in a Caron 6030 environmental chamber. Each guinea pig was placed in its own cage, and two cages were positioned on each shelf. Naïve animals were placed behind infected animals, such that the direction of airflow was toward the naïve animals. The cages used were open to airflow through the top and one side, both of which were covered by wire mesh. Although infected and exposed guinea pigs were placed in pairs, air flowed freely between shelves, allowing transmission to occur from any infected to any naïve animal.

Twenty replicate experiments were performed in which all factors remained constant except for the RH and/or temperature inside the environmental chamber. Each experiment involved eight guinea pigs, and transmission under each set of conditions was assessed in duplicate. The arrangement of animals in the environmental chamber is illustrated in Figure 1. Virus contained in nasal wash samples collected on alternating days post-inoculation (p.i.) was titrated by plaque assay to determine the infection status of each animal. Serum samples were collected from each animal prior to infection and on day 17 p.i., and seroconversion was assessed by hemagglutination inhibition assay (results in Table S1).

In general, the behavior (level of activity, food and water consumption, symptoms of infection) of guinea pigs was not observed to change with the ambient relative humidity. Likewise, animals housed at 5 °C behaved in a similar manner to those housed at 20 °C. Guinea pigs kept at 30 °C consumed more water than those housed under cooler conditions, and appeared lethargic. Consistent with our previous observations [11], influenza virus–infected guinea pigs did not display detectable symptoms of disease (e.g., weight loss, fever, sneezing, coughing) during the experiments described.

### Transmission Efficiency Is Dependent on Relative Humidity

**Figure 2:**
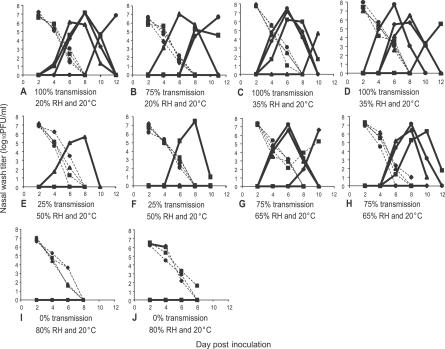
Transmission of Influenza Virus from Guinea Pig to Guinea Pig Is Dependent on Relative Humidity Titers of influenza virus in nasal wash samples are plotted as a function of day p.i. Overall transmission rate and the RH and temperature conditions of each experiment are stated underneath the graph. Titers from intranasally inoculated guinea pigs are represented as dashed lines; titers from exposed guinea pigs are shown with solid lines. Virus titrations were performed by plaque assay on Madin Darby canine kidney cells.

The results of transmission experiments performed at 20 °C and five different RHs (20%, 35%, 50%, 65%, and 80%) indicated that the efficiency of aerosol spread of influenza virus varied with RH. Transmission was highly efficient (occurred to three or four of four exposed guinea pigs) at low RH values of 20% or 35%. At an intermediate RH of 50%, however, only one of four naïve animals contracted infection. Three of four exposed guinea pigs were infected at 65% RH, while no transmission was observed at a high RH of 80% (Figure 2). Where transmission was observed, the kinetics with which infection was detected in each exposed animal varied between and within experiments. To an extent, we believe this variation is due to the stochastic nature of infection. However, while most infection events were the product of primary transmission from an inoculated animal, others could be the result of secondary transmission from a previously infected, exposed guinea pig. With the exception of the lack of transmission at 80% RH, the observed relationship between transmission and RH is similar to that between influenza virus stability in an aerosol and RH [12], suggesting that at 20 °C the sensitivity of transmission to humidity is due largely to virus stability.

### Transmission Efficiency Is Inversely Correlated with Temperature

**Figure 3:**
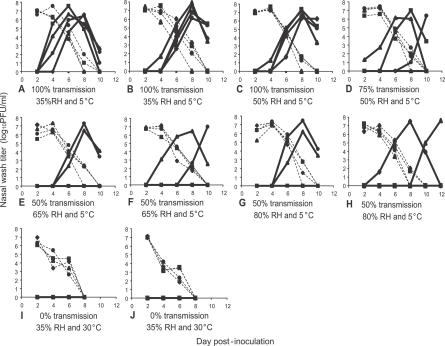
Transmission of Influenza Virus from Guinea Pig to Guinea Pig Is Highly Efficient at 5 °C and Blocked at 30 °C Titers of influenza virus in nasal wash samples are plotted as a function of day p.i. Overall transmission rate and the RH and temperature conditions of each experiment are stated underneath the graph. Titers from intranasally inoculated guinea pigs are represented as dashed lines; titers from exposed guinea pigs are shown with solid lines.

To test whether cold temperatures would increase transmission, the ambient temperature in the chamber was lowered to 5 °C and experiments were performed at 35%–80% RH. Overall, transmission was more efficient at 5 °C: 75%–100% transmission occurred at 35% and 50% RH, and 50% transmission was observed at 65% and 80% RH (Figure 3A–3H). The statistical significance of differences in transmission rates at 5 °C compared to 20 °C was assessed using the Fisher's exact test. While at 35% and 65% RH the difference was not found to be significant, at both 50% and 80% RH, transmissibility at 5 °C was found to be greater than that at 20 °C (*p* < 0.05). Conversely, when the ambient temperature was increased to 30 °C and transmission experiments carried out at a low RH of 35%, no transmission was observed (Figure 3I and 3J).

### Viral Shedding Is Increased in Animals at 5 °C

**Figure 4:**
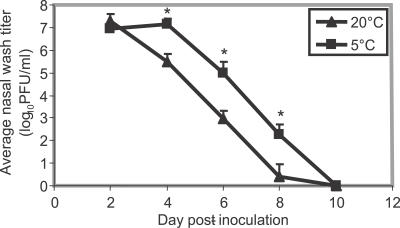
Guinea Pigs Housed at 5 °C Shed Influenza Virus at Higher Titers on Days 4, 6, and 8 p.i. Than Guinea Pigs Housed at 20 °C Average viral titers in nasal wash samples collected from animals housed at either 5 °C or 20 °C and all RH tested are plotted as function of time post-infection. Error bars indicate standard deviation; * indicates statistically significant difference between titers at 5 °C and 20 °C, with *p* ≤ 0.005.

We also observed that, although changes in RH did not affect the kinetics of viral shedding in inoculated guinea pigs, changes in temperature did. At 5 °C and all RHs tested, the intranasally inoculated guinea pigs shed higher titers of virus on days 4, 6, and 8 post-infection; most notably, peak shedding was extended in these animals by 2 d relative to guinea pigs housed at 20 °C (Figure 4). We tested whether the peak duration of viral shedding was statistically longer at 5 °C than at 20 °C by comparing the last day on which the nasal wash titer was ≥ 10^6^ plaque-forming units (PFU)/ml for each guinea pig housed at 5 °C and at 20 °C. The average of this last day value was 3.93 ± 0.63 d for animals at 5 °C and 2.21 ± 0.61 d for animals at 20 °C. By the Student's *t*-test for two independent samples, this value was significantly higher for animals housed at 5 °C than those housed at 20 °C (*p* < 0.0005). The increased duration of peak shedding at 5 °C is most likely the cause of increased transmission under cold conditions.

### Innate Immune Response Is Not Affected by Low Ambient Temperature

**Figure 5:**
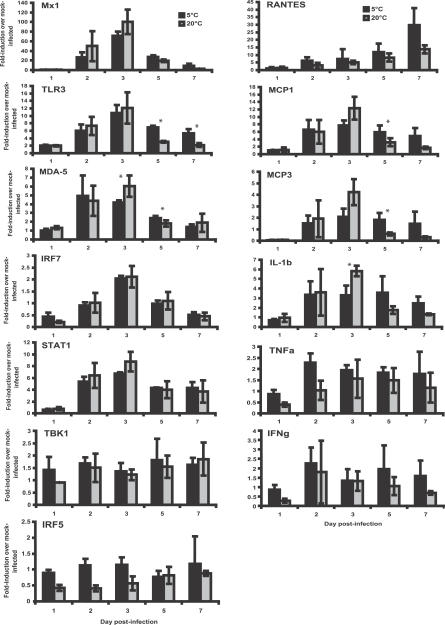
Antiviral and Pro-Inflammatory Responses Are Similar between Guinea Pigs Housed at 5 °C and 20 °C Levels of the indicated mRNA transcripts present in nasal turbinates of infected guinea pigs housed at 5 °C (black bars) or 20 °C (grey bars) were quantified by real-time PCR of reverse transcribed mRNA. RNA levels were normalized to β-actin and are expressed as fold-induction over mock-infected guinea pig. Error bars represent standard deviation. * indicates a statistically significant (*p* < 0.05) difference between 5 °C and 20 °C.

The observed differences in viral titers between animals housed at 20 °C and 5 °C suggested that cold temperature has a negative impact on host defenses early in infection. We therefore used real-time PCR to assess innate immune function in guinea pigs housed at both temperatures. The nasal turbinates were removed from three animals on each of days 1, 2, 3, 5, and 7 p.i. RNA extracted from these tissues was subjected to reverse transcription with an oligo dT primer followed by quantitative, real-time PCR with target-specific primers. Mx1, TLR3, MDA-5, IRF7, STAT1, RANTES, MCP1, MCP3, and IL-1β were found to be upregulated in response to infection in animals housed under both conditions (Figure 5). Peak expression was seen at day 3 p.i. for all of these genes except RANTES, which increased steadily up to day 7 p.i., and MDA-5 and STAT1, which showed similarly high levels of expression on days 2 and 3 p.i. While RANTES expression was elevated more in animals at 5 °C than in animals at 20 °C (*p* = 0.09), peak levels of IL-1β and MDA-5 were higher in guinea pigs at 20 °C (*p* < 0.05). TLR3, MCP1, MCP3, and MDA-5 were all expressed to significantly higher levels in animals at 5 °C than at 20 °C late in infection (day 5 and/or 7 p.i.), an observation that may simply reflect the levels of virus present at these time points. No significant upregulation was observed in either group of guinea pigs for TNFα, TBK1, IRF5, or IFNγ (Figure 5). Overall, these data indicate that innate immunity was not greatly impaired in guinea pigs housed at 5 °C relative to those at 20 °C. These findings argue against the idea that increased physiological stress experienced under cold conditions leads to a weakening of the immune response [13].

## Discussion

We have shown that the transmission of a human strain of influenza virus between guinea pigs, a highly susceptible mammalian species, is acutely sensitive to conditions of RH and temperature. Our data suggest that these two environmental factors could contribute to the seasonality of epidemic influenza.

### Mechanism of Variation in Transmission with Relative Humidity

**Figure 6:**
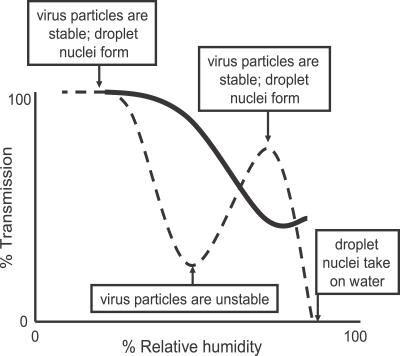
Variation of Transmission Efficiency with Relative Humidity: A Model At 20 °C (dashed line), transmission efficiency is highest at low RH, when influenza virions in an aerosol are relatively stable, and desiccation of exhaled respiratory droplets produces droplet nuclei. Transmission is diminished at intermediate RH when virus particles are relatively unstable, but improves in parallel with influenza virus stability at higher humidities. At high RH, evaporation from exhaled particles is limited, respiratory droplets settle out of the air, and transmission is blocked. At 5 °C (solid line), transmission is more efficient than at 20 °C, but is reduced to a rate of 50% at higher humidities.

Three mechanisms could potentially explain the observed influence of RH on transmission. The first acts at the level of the host: breathing dry air could cause desiccation of the nasal mucosa, leading to epithelial damage and/or reduced mucociliary clearance, which would in turn render the host more susceptible to respiratory virus infections. Long-term exposure to dry air is likely to affect influenza virus growth in the upper respiratory tract, and may indeed play a role in influenza seasonality. Nevertheless, based on the brevity of the exposure of naïve guinea pigs to dry air before becoming infected (less than 72 h), we do not believe that this mechanism played a significant role in the observed effects. The second mechanism acts at the level of the virus particle. The stability of influenza virions in an aerosol has been reported to vary with RH [12,14,15]. The most recent of these reports [12] shows viral stability to be maximal at low RH (20%–40%), minimal at intermediate RH (50%), and high at elevated RH (60%–80%). The similarity between these data on stability and our own on transmission is striking, and suggests that the stability of the virus in aerosols is a key determinant of influenza virus transmission (with the exception of the absence of transmission at high RH). The third mechanism acts at the level of the vehicle, the respiratory droplet. At low RH, evaporation of water from exhaled bioaerosols would occur rapidly, leading to the formation of droplet nuclei; conversely, at high RH, small respiratory droplets would take on water, increase in size and settle more quickly out of the air [16]. Droplet nuclei are less than 5 μm in diameter and, unlike larger droplets, they remain airborne for an extended period of time, thereby increasing the opportunity for transmission of pathogens they carry [17]. Our data suggest that, in this model system, the formation of droplet nuclei is important to transmission; we propose that at high RH (80%) exhaled respiratory droplets settle too rapidly to contribute to influenza virus spread. Based on our data, we present a model in Figure 6 of how transmission is affected by changes in RH.

### Mechanism of Improved Transmission at 5 °C

Despite the apparent fitness of animals housed at 5 °C, increased viral shedding under these conditions suggests that improved transmission at low temperature could be due to an effect on the host. This effect may act at the level of primary, physical barriers to infection. Cooling of the nasal mucosa is thought to increase the viscosity of the mucous layer and reduce the frequency of cilia beats [8]. In this way, breathing cold air would slow mucociliary clearance and thereby encourage viral spread within the respiratory tract. An alternative possibility is that, also due to cooling of the mucosal layer, virus residing in the upper airways is more stable when infected animals are kept at 5 °C. Improved persistence of released virus would increase the amount of viable virus shed, and would furthermore augment amplification of virus in the nasal passages through re-infection. The block in transmission at 30 °C and 35% RH could be explained by the opposite effect: warming of the nasal mucosa may lead to more rapid inactivation of virus particles.

### Implications for Influenza Seasonality

To our knowledge, we demonstrate for the first time that cold temperatures and low relative humidity are favorable to the spread of influenza virus. Although other factors likely contribute to the periodicity of influenza epidemics, it is clear that air temperature and RH could play an important role. Mathematical modeling indicates that only a small seasonal forcing is required to produce oscillations in infection rate of high amplitude [18]; accordingly, it is possible that the extended exposure of a small proportion of the population to outdoor winter conditions would comprise a sufficient force to create seasonal epidemics. Although the effect may therefore be small and difficult to detect, the importance of RH and temperature in the epidemiology of human influenza could be verified based on surveillance data. To this end, surveillance data with greater spatial resolution (i.e., on a regional rather than national scale) and concurrent meteorological data is needed.

The observed lack of transmission among animals housed at 30 °C raises the question of how, if our model is representative of human infection, the spread of influenza viruses occurs in tropical climates. Experiments to more closely examine this question are underway. Our preliminary data suggest that transmission at 30 °C is not improved at RHs higher than 35%. It will be interesting to test whether transmission among guinea pigs housed in the same cage (i.e., direct contact or fomite-mediated infection) is affected by RH and temperature. If transmission in this setting is not impaired at 30 °C, this may suggest that contact-based spread predominates in the tropics, whereas aerosol transmission plays a larger role in temperate climates.

Using the guinea pig model, we report a systematic analysis of the effects of RH and temperature on influenza virus transmission in a controlled setting. These data provide valuable insight into the seasonality of influenza and will aid further research into both local and global patterns of influenza virus spread within and between human populations. Our findings furthermore suggest a novel means of infection control for an important human pathogen. Influenza virus transmission indoors could potentially be curtailed by simply maintaining room air at warm temperatures (>20 °C) and either intermediate (50%) or high (80%) RHs.

## Materials and Methods

### Virus.

Influenza A/Panama/2007/99 virus (Pan/99; H3N2) was kindly supplied by Adolfo García-Sastre and was propagated in Madin Darby canine kidney cells.

### Animals.

Female Hartley strain guinea pigs weighing 300–350 g were obtained from Charles River Laboratories. Animals were allowed free access to food and water and kept on a 12-h light/dark cycle. Guinea pigs were anesthetized for the collection of blood and of nasal wash samples, using a mixture of ketamine (30 mg/kg) and xylazine (2 mg/kg), administered intramuscularly. All procedures were performed in accordance with the Institutional Animal Care and Used Committee guidelines. During guinea pig transmission experiments, strict measures were followed to prevent aberrant cross-contamination between cages: sentinel animals were handled before inoculated animals, gloves were changed between cages, and work surfaces were sanitized between guinea pigs.

### Transmission experiments.

The term “aerosol” is used herein to describe respiratory droplets of all sizes. The term “droplet nuclei” is used to refer to droplets that remain airborne (typically less than 5 μm in diameter).

Each transmission experiment involved eight guinea pigs. On day 0, four of the eight guinea pigs were inoculated intranasally with 10^3^ PFU of influenza A/Panama/2007/99 virus (150 μl per nostril in phosphate buffered saline [PBS] supplemented with 0.3% bovine serum albumin [BSA]) and housed in a separate room from the remaining animals. At 24 h p.i., each of the eight guinea pigs was placed in a “transmission cage”, a standard rat cage (Ancare R20 series) with an open wire top, which has been modified by replacing one side panel with a wire grid. The transmission cages were then placed into the environmental chamber (Caron model 6030) with two cages per shelf, such that the wire grids opposed each other (Figure 1). In this arrangement, the guinea pigs cannot come into physical contact with each other. Each infected animal was paired on a shelf with a naïve animal. The guinea pigs were housed in this way for 7 d, after which they were removed from the chamber and separated. On day 2 p.i. (day 1 post-exposure) and every second day thereafter up to day 12 p.i., nasal wash samples were collected from anesthetized guinea pigs by instilling 1 ml of PBS-BSA into the nostrils and collecting the wash in a Petri dish. Titers in nasal wash samples were determined by plaque assay of 10-fold serial dilutions on Madin Darby canine kidney cells. Serum samples were collected from each animal prior to infection and on day 17 post-infection, and seroconversion was assessed by hemagglutination inhibition assay.

All transmission experiments reported herein were performed between September 2006 and April 2007.

### Analysis of expression levels of mediators of innate immunity.

Guinea pigs were inoculated with 10^3^ PFU of Pan/99 virus intranasally and immediately housed under the appropriate conditions (5 °C or 20 °C and 35% RH). At days 1, 2, 3, 5, and 7 post-infection, three guinea pigs were killed and their nasal turbinates removed. Tissues were placed immediately in RNAlater reagent (Qiagen), and stored at 4 °C for 1 to 5 d. RNA was extracted from equivalent masses of tissue using the RNAeasy Protect Mini kit (Qiagen) and subjected to DNAse treatment (Qiagen). One microgram of RNA was subjected to reverse transcription using MMLV reverse transcriptase (Roche). One microlitre of the resultant product was used as the template in a SYBR green (Invitrogen) real-time PCR assay (Roche Light Cycler 480) with Ampli-taq Gold polymerase (Perkin-Elmer). Primers used were as follows: β-actin f AAACTGGAACGGTGAAGGTG; β-actin r CTTCCTCTGTGGAGGAGTGG; Mx1 f CATCCCYTTGrTCATCCAGT; Mx1 r CATCCCyTTGRTCATCCAGT; MDA-5 f GAGCCAGAGCTGATGARAGC; MDA-5 r TCTTATGWGCATACTCCTCTGG; IL-1β f GAAGAAGAGCCCATCGTCTG; IL-1β r CATGGGTCAGACAACACCAG; RANTES f GCAATGCTAGCAGCTTCTCC; RANTES r TTGCCTTGAAAGATGTGCTG; TLR3 f TAACCACGCACTCTGTTTGC; TLR3 r ACAGTATTGCGGGATCCAAG; TNFα f TTCCGGGCAGATCTACTTTG; TNFα r TGAACCAGGAGAAGGTGAGG; MCP-1 f ATTGCCAAACTGGACCAGAG; MCP-1 r CTACGGTTCTTGGGGTCTTG; MCP-3 f TCATTGCAGTCCTTCTGTGC; MCP-3 r TAGTCTCTGCACCCGAATCC; IFNγ f GACCTGAGCAAGACCCTGAG; IFNγ r TGGCTCAGAATGCAGAGATG; STAT1 f AAGGGGCCATCACATTCAC; STAT1 r GCTTCCTTTGGCCTGGAG; TBK1 f CAAGAAACTyTGCCwCAGAAA; TBK1 r AGGCCACCATCCAykGTTA; IRF5 f CAAACCCCGaGAGAAGAAG; IRF5 r CTGCTGGGACtGCCAGA; IRF7 f TGCAAGGTGTACTGGGAGGT; IRF7 r TCACCAGGATCAGGGTCTTC (where R = A or G, Y = C or T, W = A or T, K = T or G). Primer sequences were based either on guinea pig mRNA sequences available in GenBank (MCP1, MCP3, IL-1b, IFNγ, RANTES, TLR3, TNFα, and β-actin), or on the consensus sequence of all species available in GenBank (Mx1, MDA-5, IRF5, IRF7, STAT1, and TBK1). Sequencing of each PCR product indicated that all primer pairs were specific for the expected transcript. Reactions were performed in duplicate and normalized by dividing the mean value of the cycle threshold (Ct) of β-actin expressed as an exponent of 2 (2^Ct^) by the mean value of 2^Ct^ for the target gene. The fold-induction over the mock-infected was then calculated by dividing the normalized value by the normalized mock value. Data is represented in Figure 5 as the mean of three like samples (nasal turbinates harvested on the same day p.i. from three guinea pigs) ± standard deviation.

### Statistical analyses.

Statistical analyses were performed using GraphPad Prism 5 software.

## Supporting Information

Table S1 Seroconversion of Inoculated and Exposed Guinea Pigs Results of hemagglutination inhibition tests for each transmission experiment are shown.
(58 KB DOC)

### Accession Numbers

The GenBank (http://www.ncbi.nlm.nih.gov/Genbank/index.html) accession numbers of guinea pig genes used for primer design are as follows: β-actin (AF508792.1); IFNγ (AY151287.1); IL-1β (AF119622); MCP-1 (L04985); MCP-3 (AB014340); RANTES (CPU77037); TLR3 (DQ415679.1); and TNFα (CPU77036).

## Abbreviations

Ct: cycle threshold
p.i.: post-inoculation
PFU: plaque-forming units
RH: relative humidity

## References

[ppat-0030151-b001] Thompson WW, Shay DK, Weintraub E, Brammer L, Bridges CB (2004). Influenza-associated hospitalizations in the United States. JAMA.

[ppat-0030151-b002] Viboud C, Alonso WJ, Simonsen L (2006). Influenza in tropical regions. PLoS Med.

[ppat-0030151-b003] Shek LP, Lee BW (2003). Epidemiology and seasonality of respiratory tract virus infections in the tropics. Paediatr Respir Rev.

[ppat-0030151-b004] Lofgren E, Fefferman N, Naumov YN, Gorski J, Naumova EN (2007). Influenza seasonality: underlying causes and modeling theories. J Virol.

[ppat-0030151-b005] Dowell SF (2001). Seasonal variation in host susceptibility and cycles of certain infectious diseases. Emerg Infect Dis.

[ppat-0030151-b006] Cannell JJ, Vieth R, Umhau JC, Holick MF, Grant WB (2006). Epidemic influenza and vitamin D. Epidemiol Infect.

[ppat-0030151-b007] Brownstein JS, Wolfe CJ, Mandl KD (2006). Empirical evidence for the effect of airline travel on inter-regional influenza spread in the United States. PLoS Med.

[ppat-0030151-b008] Eccles R (2002). An explanation for the seasonality of acute upper respiratory tract viral infections. Acta Otolaryngol.

[ppat-0030151-b009] Hammond GW, Raddatz RL, Gelskey DE (1989). Impact of atmospheric dispersion and transport of viral aerosols on the epidemiology of influenza. Rev Infect Dis.

[ppat-0030151-b010] Schulman JL, Kilbourne ED (1963). Transmission of influenza virus infection in mice. II. Some factors affecting the incidence of transmitted infection. J Exp Med.

[ppat-0030151-b011] Lowen AC, Mubareka S, Tumpey TM, García-Sastre A, Palese P (2006). The guinea pig as a transmission model for human influenza viruses. Proc Natl Acad Sci U S A.

[ppat-0030151-b012] Schaffer FL, Soergel ME, Straube DC (1976). Survival of airborne influenza virus: effects of propagating host, relative humidity, and composition of spray fluids. Arch Virol.

[ppat-0030151-b013] Shephard RJ, Shek PN (1998). Cold exposure and immune function. Can J Physiol Pharmacol.

[ppat-0030151-b014] Harper GJ (1961). Airborne micro-organisms: survival tests with four viruses. J Hyg (Lond).

[ppat-0030151-b015] Hemmes JH, Winkler KC, Kool SM (1960). Virus survival as a seasonal factor in influenza and poliomyelitis. Nature.

[ppat-0030151-b016] Tellier R (2006). Review of aerosol transmission of influenza A virus. Emerg Infect Dis.

[ppat-0030151-b017] Buxton Bridges C, Kuehnert MJ, Hall CB (2003). Transmission of influenza: implications for control in health care settings. Clin Infect Dis.

[ppat-0030151-b018] Dushoff J, Plotkin JB, Levin SA, Earn DJ (2004). Dynamical resonance can account for seasonality of influenza epidemics. Proc Natl Acad Sci U S A.

