# Supplementary material for: Influenza Virus Transmission Is Dependent on Relative Humidity and Temperature
Source: PLoS Pathog. 2007 Oct 19;3(10):e151. doi: 10.1371/journal.ppat.0030151 (PMC2034399; doi:10.1371/journal.ppat.0030151)
Supplement: Supplementary file 1 [file ppat.0030151.st001.doc]

**Supplementary Table 1. Seroconversion of inoculated and exposed guinea pigs**

| Experiment* | Temperature (°C) | Relative Humidity (%) | Seroconversion† | |
| --- | --- | --- | --- | --- |
|  |  |  | Inoculated | Exposed |
| 1A | 20 | 20 | 1 (16×)  2 (16×)  3 (16×)  4 (8×) | 5 (4×)  6 (4×)  7 (2×)  8 (4×) |
| 1B | 20 | 20 | 1 (16×)  2 (16×)  3 (32×)  4 (32×) | 5 (4×)  6 (16×)  7 (4×)  8 - |
| 1C | 20 | 35 | 1 (8×)  2 (16×)  3 (16×)  4 (16×) | 5 (4×)  6 (4×)  7 (2×)  8 (4×) |
| 1D | 20 | 35 | 1 n.d.  2 (16×)  3 (32×)  4 (32×) | 5 (2×)  6 (8×)  7 (8×)  8 n.d. |
| 1E | 20 | 50 | 1 (8×)  2 (32×)  3 (32×)  4 (16×) | 5 -  6 (4×)  7 -  8 (2×) |
| 1F | 20 | 50 | 1 (8×)  2 (16×)  3 (16×)  4 (16×) | 5 (8×)  6 (2×)  7 (2×)  8 (2×) |
| 1G | 20 | 65 | 1 (32×)  2 (16×)  3 (8×)  4 (32×) | 5 -  6 (32×)  7 (16×)  8 (8×) |
| 1H | 20 | 65 | 1 (16×)  2 (16×)  3 (16×)  4 (16×) | 5 (4×)  6 (16×)  7 (8×)  8 (2×) |
| 1I | 20 | 80 | 1 (16×)  2 (16×)  3 (8×)  4 (32×) | 5 -  6 -  7 -  8 - |
| 1J | 20 | 80 | 1 (4×)  2 n.d.  3 (8×)  4 (8×) | 5 -  6 -  7 -  8 - |
| 2A | 5 | 35 | 1 (32×)  2 (8×)  3 (8×)  4 (16×) | 5 (32×)  6 (32×)  7 (16×)  8 (16×) |
| 2B | 5 | 35 | 1 (32×)  2 (8×)  3 (16×)  4 (16×) | 5 (8×)  6 (8×)  7 (4×)  8 (16×) |
| 2C | 5 | 50 | 1 (32×)  2 (16×)  3 (32×)  4 (32×) | 5 (16×)  6 (16×)  7 (16×)  8 (8×) |
| 2D | 5 | 50 | 1 (32×)  2 (32×)  3 (64×)  4 (32×) | 5 (8×)  6 (16×)  7 (8×)  8 (2×) |
| 2E | 5 | 65 | 1 (32×)  2 (16×)  3 (32×)  4 (32×) | 5 -  6 (16×)  7 (32×)  8 - |
| 2F | 5 | 65 | 1 (16×)  2 (16×)  3 (64×)  4 n.d. | 5 -  6 (16×)  7 (8×)  8 - |
| 2G | 5 | 80 | 1 (16×)  2 (32×)  3 (32×)  4 (32×) | 5 (2×)  6 (16×)  7 (2×)  8 (16×) |
| 2H | 5 | 80 | 1 (16×)  2 (32×)  3 (32×)  4 (32×) | 5 (2×)  6 (16×)  7 (2×)  8 (16×) |
| 2I | 30 | 35 | 1 (8×)  2 (32×)  3 (16×)  4 (16×) | 5 -  6 (2×)  7 (4×)  8 - |
| 2J | 30 | 35 | 1 (4×)  2 (16×)  3 (32×)  4 (8×) | 5 (2×)  6 -  7 -  8 - |

* The letter of each experiment refers to that used in Fig.1 or Fig.2

† Guinea pigs numbered 1 to 4 were intranasally inoculated; guinea pigs numbered 5 to 8 were exposed to infected animals. “n.d.” indicates that terminal serum was not collected because guinea pig died prior to day 17 post-infection. “-” indicates that no change in hemagglutination inhibition activity was detected.
